# Cognitive Impairment Risk in Subjective Cognitive Decline: Roles of Sleep, Physical Frailty, and Their Interaction

**DOI:** 10.1093/geroni/igaf122.1962

**Published:** 2025-12-31

**Authors:** Jing Huang, Katie Stone, Chakra Budhathoki, Junxin Li

**Affiliations:** Johns Hopkins University, Baltimore, Maryland, United States; California Pacific Medical Center Research Institute, Castro Valley, California, United States; Johns Hopkins University, Baltimore, Maryland, United States; Johns Hopkins University, Baltimore, Maryland, United States

## Abstract

Subjective cognitive decline (SCD)—a self-perceived decline in cognition without objective impairment—may signal early Alzheimer’s disease or dementia. Sleep disturbances and physical frailty are linked to cognitive decline, but their roles in the progression from SCD to objective cognitive impairment and dementia, and their potential interaction, remain unclear. This study examined the associations of sleep disturbance, physical frailty, and their interaction with incident cognitive impairment and dementia over a 10-year follow-up period using six waves of Health and Retirement Study (HRS) data (2010–2020). While analysis on sleep used all 3,014 individuals with SCD at baseline, analyses involving frailty data included a subsample of 1,141. Sleep disturbance was assessed using the modified Jenkins Sleep Scale, and physical prefrailty/frailty was defined by the Fried frailty phenotype. Cognitive impairment and dementia incidence were determined using validated HRS cognitive cutoffs. Discrete-time survival analyses, adjusted for sociodemographic and health factors, found no independent association between baseline sleep disturbance and subsequent cognitive impairment or dementia. However, physical prefrailty/frailty was significantly associated with increased risk of cognitive impairment (adjusted odds ratio [aOR] = 1.44, 95% CI: 1.15–1.80) and dementia (aOR = 1.89, 95% CI: 1.23–2.93). A significant interaction (aOR = 0.90, 95% CI: 0.81–1.00, p < 0.05) suggested that sleep disturbance was more strongly associated with cognitive impairment in non-frail older adults than in those who were prefrail or frail. In conclusion, physical prefrailty/frailty is a significant predictor of cognitive impairment in older adults with SCD, while the effect of sleep disturbance may vary by frailty status.

